# Controle da Pressão Arterial: O Segredo é... Trabalho em Equipe!

**DOI:** 10.36660/abc.20200544

**Published:** 2020-08-19

**Authors:** Andrea Pio-Abreu, Luciano F. Drager

**Affiliations:** 1 Divisão de Nefrologia Faculdade de Medicina Universidade de São Paulo São Paulo SP Brasil Unidade de Hipertensão, Divisão de Nefrologia, Faculdade de Medicina da Universidade de São Paulo,São Paulo, SP - Brasil; 2 Instituto do Coração Faculdade de Medicina Universidade de São Paulo São Paulo SP Brasil Unidade de Hipertensão, Instituto do Coração (InCor), Faculdade de Medicina da Universidade de São Paulo,São Paulo, SP – Brasil

**Keywords:** Hipertensão, Pressão Arterial, Prevenção e Controle, Fatores de Risco, Equipe de Assistência ao Paciente/tendências, Anti-Hipertensivos, Adesão à Medicação

A hipertensão é uma das principais causas de morte cardiovascular.^1^ De fato, dados do *Heart and Stroke Statistics* mostraram que 45% da mortalidade cardiovascular é potencialmente atribuída à hipertensão.^1^ Esse cenário preocupante não mudou nas últimas décadas, apesar da disponibilidade das intervenções não-farmacológicas e o desenvolvimento de várias classes de remédios anti-hipertensivos que efetivamente contribuíram para o controle da pressão arterial (PA).^2 - 5^ As razões de nossa baixa efetividade no controle da PA no nível populacional são múltiplas, incluindo a falta de políticas públicas organizadas que regulem o consumo de sal e aumentem a conscientização, a detecção precoce e o tratamento efetivo. Desafios adicionais incluem a característica assintomática da hipertensão, inércia terapêutica, entre outros.^1^

Nesta edição dos Arquivos Brasileiros de Cardiologia, Jardim et al.,^6^ relataram dados de um estudo retrospectivo explorando uma estratégia de equipe multidisciplinar na taxa de controle da pressão arterial (estabelecida no tradicional <140/90 mmHg). Os autores avaliaram dados demográficos e clínicos de 1.548 pacientes hipertensos de um centro especializado em hipertensão, acompanhados regularmente por 7,6 ± 7,1 anos (média de idade de 62 anos, 73,6% de mulheres).

A abordagem multidisciplinar descrita pelos autores consistiu na disponibilidade de enfermeiros, nutricionistas, terapeutas ocupacionais, educadores físicos, psicólogos e musicoterapeutas trabalhando em conjunto com os médicos da equipe (clínicos gerais, cardiologistas, endocrinologistas e nefrologistas). O intervalo máximo para consultas médicas foi de 3 meses. De acordo com as necessidades dos pacientes (determinados pela avaliação clínica), os médicos agendavam visitas aos profissionais mencionados acima em uma demanda flexível. Além disso, foram realizadas atividades educacionais e de promoção da saúde a cada duas semanas com os pacientes. Toda essa informação foi registrada em um formulário padronizado. Usando essa estratégia, os autores descobriram que essa abordagem de equipe multidisciplinar estava associada a um controle geral da pressão arterial de 68%, sendo mais acentuado naqueles com idade ≥60 anos (OR 1,45; IC 95% [1,13-1,90]) e em mulheres (OR 1,36; IC95% [1,09-1,88]). Por outro lado, pacientes com diabetes foram associados a uma menor probabilidade de atingir a meta de PA em comparação com pacientes sem diabetes. Curiosamente, não foram observadas diferenças significativas no número de medicamentos anti-hipertensivos nos grupos que controlaram ou não a PA. Esse achado sugere que a adesão a essa abordagem multidisciplinar pode variar, como geralmente observado em qualquer outra intervenção.

Vale ressaltar o mérito do serviço relacionado, cuja prática multidisciplinar é adotada, de acordo com os autores, há mais de 25 anos.^6^ O controle da PA é impressionante, considerando as estimativas atuais do controle da PA no Brasil (geralmente abaixo de 30% em estudos individuais).^3^ De maneira geral, a principal contribuição dessa investigação é que a literatura é relativamente escassa (principalmente de grandes estudos multicêntricos observacionais ou randomizados) ao abordar o impacto potencial de uma equipe multidisciplinar em pacientes com hipertensão. Estudos anteriores envolvendo tamanhos de amostras modestos sugeriram a importância dos enfermeiros na melhora da adesão a tratamentos anti-hipertensivos e do efeito de jaleco branco.^6 - 11^ Da mesma forma, a abordagem ativa de farmacêuticos, educadores físicos e nutricionistas parece contribuir para melhorar a adesão e o controle da PA.^12 , 13^ Entretanto, é crucial definir se toda a estrutura de equipe disponível (e não os ‘compartimentos’ distintos) pode contribuir para a eficácia do controle da PA. Em outras palavras, o todo é melhor do que qualquer componente individual ou a soma das partes?

O estudo realizado por Jardim et al. não foi projetado para abordar essa questão, mas destacou que a luta contra a hipertensão não se baseia em um único ator. Infelizmente, a falta de um grupo controle (por exemplo, pacientes de outros centros sem acesso a uma abordagem de equipe multidisciplinar organizada) e o desenho retrospectivo impedem conclusões definitivas, mas abrem caminho para futuras investigações nesta importante área de pesquisa. Atenção especial deve ser dedicada aos pacientes com diabetes. A menor taxa de controle da PA nos desafia a realizar esforços extras nessa população de alto risco cardiovascular.
