# Supplementary material for: Controle da Pressão Arterial: O Segredo é... Trabalho em Equipe!
Source: Arq Bras Cardiol. 2020 Aug 19;115(2):182–3. [Article in Portuguese] doi: 10.36660/abc.20200544 (PMC8384293; doi:10.36660/abc.20200544)
Supplement: Translation

# Translation: Blood Pressure Control: The secret is…Team Work!

**Authors:** Andrea Pio-Abreu, Luciano F. Drager

**Affiliations:** 1 Brazil Unidade de Hipertensão, Divisão de Nefrologia, Faculdade de Medicina da Universidade de São Paulo, São Paulo, SP - Brazil; 2 Brazil Unidade de Hipertensão, Instituto do Coração (InCor), Faculdade de Medicina da Universidade de São Paulo, São Paulo, SP – Brazil

**Keywords:** Hypertension, Blood Pressure, Prevention and Control, Risk Factors, Patient Care Team/trends, Antihypertensive Agents, Medication Adherence

Hypertension is one of the leading causes of cardiovascular death.^1^ Indeed, data from the Heart and Stroke Statistics showed that 45% of the cardiovascular mortality is potentially attributed to hypertension.^1^ This worrying scenario has not been changing in the last decades despite the availability of non-pharmacological interventions and the development of several anti-hypertensive classes that effectively contributed to blood pressure (BP) control.^2 - 5^ The reasons for our low effectiveness in controlling BP at the population level are multiple, including the lack of organized public policies regulating salt consumption and increasing awareness, early detection, and effective treatment. Additional challenges include the asymptomatic characteristic of hypertension, therapeutic inertia, among others.^1^

In this edition of the *Arquivos Brasileiros de Cardiologia* , Jardim et al.^6^ reported data from a retrospective study exploring a multidisciplinary team strategy in the rate of BP control (set at the traditional <140/90 mmHg). The authors evaluated demographic and clinical data of 1548 hypertensive patients from a specialized hypertension center who have been followed up regularly for 7.6 ± 7.1 years (mean age 62 years, 73.6% women). The multidisciplinary approach described by the authors consisted by the availability of nurses, nutritionists, occupational therapists, physical educators, psychologists and music therapists working in conjunction with the staff physicians (general practitioners, cardiologists, endocrinologists, and nephrologists). The maximal interval for medical outpatient visits was 3 months. According to the patients’ needs (determined by the clinical evaluation), the physicians scheduled visits to the professionals mentioned above in a flexible demand. In addition, educational and health promotion activities were performed every two weeks with patients. All this information was recorded on a standardized form. Using this strategy, the authors found that this multidisciplinary team approach was associated with an overall 68% BP control, being more prominent in those aged ≥60 years (OR 1.45; 95% CI [1.13-1.90]), and females (OR 1.36; 95% CI [1.09-1.88]). In contrast, patients with diabetes were associated with a lower probability of reaching the BP target compared to patients without diabetes. Interestingly, no significant differences in the number of anti-hypertensive drugs were observed in the groups who controlled or not BP. This finding suggests that the adherence of this multidisciplinary approach may vary as usually observed with any other intervention.

It is worth mentioning the merit of the related service, whose multidisciplinary practice has been adopted, according to the authors, for over 25 years.^6^ The BP control is impressive, considering the current estimates of BP control in Brazil (usually lower than 30% in individual studies).^3^ Overall, this investigation’s major contribution is that the literature is relatively scarce (particularly from large multicenter observational or randomized studies) in addressing the potential impact of a multidisciplinary team in patients with hypertension. Previous studies involving modest sample sizes suggested the importance of nurses in improving adherence to anti-hypertensive treatments and white coat-effect.^6 - 11^ Similarly, the active approach from pharmacists, physical educators, and nutritionists seem to contribute to improving adherence and BP control.^12 , 13^ However, it is crucial to define if the whole team structure available (and not distinct ‘compartments’) may contribute to the effectiveness of BP control. In other words, is the whole better than any individual component or the sum of the parts? The study conducted by Jardim et al. was not designed to address this question but highlighted that the fight against hypertension is not based on a single actor. Unfortunately, the lack of a control group (for instance, patients from other centers with no access to an organized multidisciplinary team approach) and the retrospective design prevent any definitive conclusions but pave the way for future investigations in this important research area. Particular attention should be devoted to patients with diabetes. The lower rate of BP control challenging us for extra efforts for this high cardiovascular risk population.
